# A Review on the Extraction of Quantitative Retinal Microvascular Image Feature

**DOI:** 10.1155/2018/4019538

**Published:** 2018-07-02

**Authors:** Kuryati Kipli, Mohammed Enamul Hoque, Lik Thai Lim, Muhammad Hamdi Mahmood, Siti Kudnie Sahari, Rohana Sapawi, Nordiana Rajaee, Annie Joseph

**Affiliations:** ^1^Department of Electrical and Electronics Engineering, University Malaysia Sarawak (UNIMAS), 94300 Kota Samarahan, Kuching, Malaysia; ^2^Department of Ophthalmology, Faculty of Medicine and Health Sciences (FMHS), University Malaysia Sarawak (UNIMAS), 94300 Kota Samarahan, Sarawak, Malaysia; ^3^Department of Para-Clinical Sciences, Faculty of Medicine and Health Sciences (FMHS), University Malaysia Sarawak (UNIMAS), 94300 Kota Samarahan, Sarawak, Malaysia

## Abstract

Digital image processing is one of the most widely used computer vision technologies in biomedical engineering. In the present modern ophthalmological practice, biomarkers analysis through digital fundus image processing analysis greatly contributes to vision science. This further facilitates developments in medical imaging, enabling this robust technology to attain extensive scopes in biomedical engineering platform. Various diagnostic techniques are used to analyze retinal microvasculature image to enable geometric features measurements such as vessel tortuosity, branching angles, branching coefficient, vessel diameter, and fractal dimension. These extracted markers or characterized fundus digital image features provide insights and relates quantitative retinal vascular topography abnormalities to various pathologies such as diabetic retinopathy, macular degeneration, hypertensive retinopathy, transient ischemic attack, neovascular glaucoma, and cardiovascular diseases. Apart from that, this noninvasive research tool is automated, allowing it to be used in large-scale screening programs, and all are described in this present review paper. This paper will also review recent research on the image processing-based extraction techniques of the quantitative retinal microvascular feature. It mainly focuses on features associated with the early symptom of transient ischemic attack or sharp stroke.

## 1. Introduction

One of the most important subfields of biomedical engineering is the analysis of fundus retinal images. Analysis of the human fundus eye images has become the key point for diagnosing the various pathologies of retinal vasculature. Furthermore, image analysis provides a simple and noninvasive visualization of the retinal blood vessels in those high risk ophthalmologic medical conditions [1–3].

The fundus retinal images are directly captured from human eye that includes some other landmarks like microcirculation system of the retina, macula, optic disc, fovea, microaneurysm, and exudates [4]. This cost-effective, simple image acquisition system can be used in the large-scale screening programs and retinal image analysis developing mathematical and computational techniques. In addition, physicians can benefit from this technique as to objectively assess abnormal symptoms such as vessel tortuosity, vessel width, bifurcation angles, branching angles, and vessel caliber. All these features are useful for early detection of hypertensive and diabetic retinopathy, macular degeneration, acute stroke, neovascular glaucoma, and some other cardiovascular diseases [1, 3, 5–9].

Some distinct changes in the retinal microvasculature are recognized as the preindicator of subsequent vascular incidents like ischemic stroke or acute stroke [10]. It was found in many types of research that there is a clear relationship between the ocular funduscopic abnormalities and acute stroke even though the blood pressure and other vascular risk factors are in control [11]. According to the study of [12] with a multiethnic cohort, retinal arteriolar narrowing and retinopathy of diabetic free people have an association with increased risk of acute stroke. But the Cardiovascular Health Study stated that there is no association between retinal arteriolar caliber (diameter) and stroke but rather there is a close association between stroke and the larger venular caliber (diameter) [13]. Rotterdam cohort study also came into a decision after a long-term observation that the retinal venular diameter is associated with any stroke or ischemic stroke [14].

The authors of [15] examined the association of hypertensive retinopathy with the risk of stroke in their population base study. Retinal microvascular abnormalities like microaneurysm, arteriovenous nicking, haemorrhages, and vessel caliber are considered as associative to the stroke and indicative of death from stroke and IHD (Ischemic Heart Diseases) [1]. A population-based study collaborating with Beaver Dam Eye Study (BDES) of [1] revealed that increased diameter ratio was associated with increased stroke mortality (P = 0.02 unadjusted). The paper [16] searched MEDLINE and EMBASE to find out the relation between microvascular changes of retinal microvasculature and prevalence or incident of stroke. In the study of [16] 20659 patients were involved, 1178 patients of them had stroke, and they found the Odd Ratio (OR) of stroke and retinal arteriolar narrowing and AV nicking was 1.42 and 1.91, respectively, which indicated that these abnormalities are slightly associated with stroke. Microvascular lesions like microaneurysm and haemorrhages which were found as highly associated with stroke as their OR were 3.83 and 3.21, respectively, and the OR between stroke and arteriole narrowing and venular network are 2.28 and 1.80 indicated the association of these abnormalities with stroke [16]. A cohort study of Asian Malay persons consisting of 3189 patients, free from prevalent stroke at baseline, revealed that 51 (1.93%) participants had an incident stroke event that could be predicted by analyzing the microvascular changes of retinal vasculature [17]. Prevalent and incident stroke have the association with retinopathy and venular widening [18]. Retinal vessel widening is also the predictor of hypertensive retinopathy which also has an association with stroke [19].

Many techniques have been developed using image processing principle to measure the vessel diameter of retinal microvasculature. Suppose some of the available vessel diameter measurement techniques are based on Linear Discriminant Analysis (LDA), image gradient segmentation technique (ARG) for vessel edge detection, using active contour [20–22], mask creation [20], graph-theoretic method [23, 24] Multistep Regression Method (Higher order Gaussian modeling) [25], Adaptive Higuchi's Dimension [26], and so on.

There are several datasets for fundus retinal images that are publicly available for the researchers from all over the world. High-Resolution Fundus Image Database (HRFID) and Digital Retinal Images for Vessel Extraction (DRIVE) are two kinds of publicly open datasets that can be accessed by the researchers [6, 27]. Researchers from digital image processing discipline can use images from these datasets as the raw materials for their experiment. REVIEW (Retinal Vessel Image set for Estimation of Width) is another publicly available standard dataset that includes various normal and pathological retinal images for the evaluation of the algorithm for retinal vessel diameter measurement. REVIEW dataset includes 16 images with 193 vessel segments that demonstrate the different types of pathologies and vessel types and this dataset consists of four subsets which are HRIS (High-Resolution Image Set), VDIS (Vascular Disease Image), CLRIS (Central Light Reflex Image Set), and KPIS (Kick Point Image) [28].

This review is focused mainly on the retinal vessel diameter measurement algorithms, applications of image processing technique, to provide an overview of the recent advancement in measuring the diameter of the retinal blood vessel. In biomedical engineering, the existing retinal image analysis methods are still dependent on a bulk of human observers. The blood vessel diameter measurement methods are also not fully automated. There is limited published work on automation of blood vessel diameter measurement. For medical diagnosing systems, maximum precision is a must for detecting the diseases as it is one of the most sensitive issues for proper diagnosing. This review is a prestep of our future work of shaping a novel, automated, and computer-aided algorithm to measure the diameter of the retinal blood vessel with maximum accuracy.

In the following sections of this paper, we briefly discussed the abnormalities of retinal microvascular features, responsible for stroke and the circumstantial scenario of the existing methods for analyzing these abnormalities. Here we emphasized to manifest especially the retinal blood vessel diameter measurement techniques as it is one of the most important markers of prevalent and incident stroke. The basic principle of digital image processing and a general digital image processing procedure for extracting the features of the retinal image containing image acquisition, grey-scale image, image enhancement, restoration, segmentation, registration and vessel extraction subsection are also described in this paper in a cabalistic manner. The arising challenges of present retinal image processing techniques for measuring the blood vessel diameter and the future scopes of this field are also explained in this paper.

## 2. Retinal Vascularization

**Figure 1:**
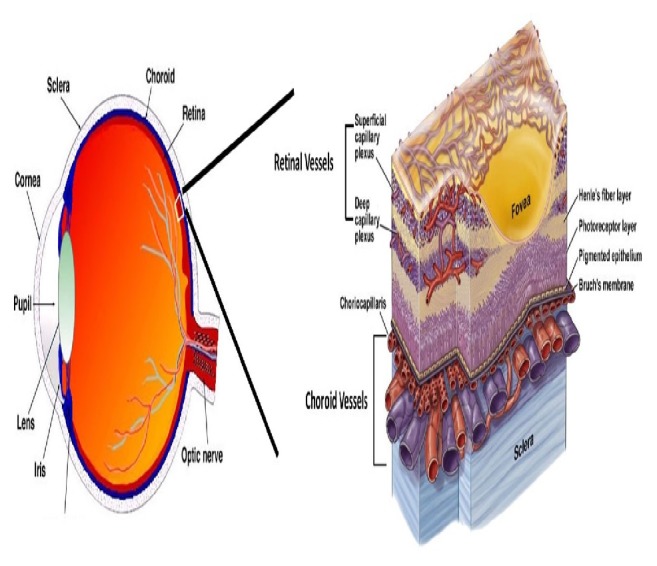
Schematic diagram of the retinal vasculature adapted from [29].

The retina is a highly vascular tissue, which receives supply from two sources, namely, central and choroidal circulatory system (Figure 1). The central blood vessel supplies the inner retina which made ~30% of the retinal blood flow [40]. Central retinal artery (CRA) runs through the optic disc and enters the inner layer of the retina. CRA branches into superior- and inferior- arteries with diameters of 150*μ*m [41]. Ultimately it forms a network of capillaries with diameters of 5*μ*m [42]. On the other hand, blood from the retina drains into the central retinal vein (CRV). Approximately 70% of the retinal blood flow is supplied by the choroidal blood vessel which nourishes the outer retina and the photoreceptor-retinal pigment epithelium (RPE) complex located adjacent to Bruch's membrane [40]. Apart from nourishing the region, the choroidal circulatory system serves as a heat sink which removes generated metabolic heat due to light photons strike on the photopigments and the melanin of the RPE [43]. Figure 1 shows the schematic diagram of the retinal vasculature.

### 2.1. Pathology of Retinal Vasculature

There is a range of eye diseases particularly affecting retinal blood vessel. Among determinant factors of retinal vascular disorders are a physiological hardening of the artery walls in aging and existing vascular diseases such as high blood pressure and atherosclerosis. Most common retinal vascular disorders are hypertensive retinopathy, Retinal Vein Occlusion (RVO), Central Retinal Artery Occlusion (CRAO), and Diabetic Retinopathy. Monitoring of the retinal vasculature through retinal imaging and vascular caliber monitoring permit a direct assessment of the retinal vascular disorders.

### 2.2. Hypertensive Retinopathy

Hypertensive retinopathy occurs when arterioles and venules of the retina are damaged, which eventually leads to blindness. Thus, several classification systems have been developed to facilitate early identification [44]. Hypertensive retinopathy initially affects the retinal blood vessels at all tributary levels, especially the arterial vessels. This process is known as sclerosis (thickening and stiffening of the artery), which can alter the angular course of the artery and may also affect the tributary angles. Furthermore, the artery and vein junction share a common adventitial sheath. Any sclerotic changes to the artery in this enclosed common “compartment” space can further compress the weaker venular vessels, which can result in further changes to the angular course of the veins as well [45]. Such angular changes can be further studied to determine its significance in the prediction and assessment of disease process and progression.

### 2.3. Retinal Vein Occlusion (RVO)

Retinal Vein Occlusion (RVO) disorder is commonly associated with various underlying systemic disorders including arterial hypertension, diabetes mellitus, dyslipidemia, and systemic vasculitis. RVO may be found in varying blood vessel caliber. This includes central retinal vein occlusion (CRVO); media such as hemicentral retinal vein occlusion and small-caliber veins such as branch retinal vein occlusion (BRVO) [46].

Retinal vein occlusion can be divided into central and branch. The branch retinal vein occlusion can happen at the various tributary levels, especially the first and second tributaries. However, prior to a full-blown vein occlusion, a temporary phase known as impending retinal vein occlusion may occur. In this phase, the affected veins will become more engorged, dilated, and tortuous, and the course and tributary angle of the affected veins will alter with this pathological process [50]. These angular changes can be further studied to determine its significance in the prediction and assessment of disease progression.

### 2.4. Central Retinal Artery Occlusion (CRAO)

Central Retinal Artery Occlusion (CRAO) is an ophthalmic emergency analogous to the acute stroke of the eye. Unfortunately, presentation of such cases to the appropriate medical attention passed the stage of reversibility. The clinical presentation with the central artery occluded is invariably a pale retina with a central “cherry red spot,” as the retina is devoid of oxygenated blood. It signifies end-organ ischemia and often the underlying atherosclerotic disease [55].

### 2.5. Diabetic Retinopathy

One-third of people with diabetes would have diabetic retinopathy (DR). This condition is ranked as the fifth most common cause of blindness worldwide [56]. DR is strongly associated with a prolonged duration of diabetes, hyperglycaemia, and hypertension. Severe stages of DR include proliferative DR, caused by vascular leakage and new retinal blood vessels growth amplified by chemokine secretion such as vasculoendothelial growth factor (VEGF) and diabetic macular oedema, in which there are exudation and oedema in the central part of the retina [57, 58]. At present, intraocular treatment modalities for diabetic eye disease include laser photocoagulation, intravitreous injections of anti-VEGF and steroid agents, and vitreoretinal surgery [58].

The pathophysiology of diabetic retinopathy initially affects the retinal blood vessels. Due to poor glycaemic control, the advanced glycation end products (AGEs) will invariably damage the pericytes which are the cellular supporting structure of the retinal capillaries. This leads to weaker and hence leaky capillaries and promoting microhypertension of the retinal blood vessels. This, in turn, will result in various retinal vascular changes including dilated, tortuous vessels with altering course and tributary angles [59]. This angular change can be further studied to determine its significance in the prediction and assessment of disease progression.

## 3. The Image Processing Techniques for Analyzing and Detecting the Abnormal Features of the Retinal Vasculature in Stroke

The microvascular structure of retinal blood vessel plays a significant role in diagnosing the possibility of causing stroke. Researchers found from population-based studies that diabetic retinopathy signs like microaneurysm and hard exudates and the hypertensive retinopathy signs like arteriovenous nicking and focal retinal arteriolar narrowing were also associated with the acute stroke and stroke mortality even if the people were free from other stroke risk factors [19, 48].

A cohort study in [49] investigated that the most retinal microvascular features that predicted incident stroke and ischemic stroke and that adjusted relative risk with any retinopathies are microaneurysm, soft exudates, bolt haemorrhages, flame-shaped haemorrhages, arteriovenous (AV) nicking, and decreasing Artery to Vein Ratio (AVR) [19, 49, 51, 60, 61].

### 3.1. AV Nicking Observation

**Figure 2:**
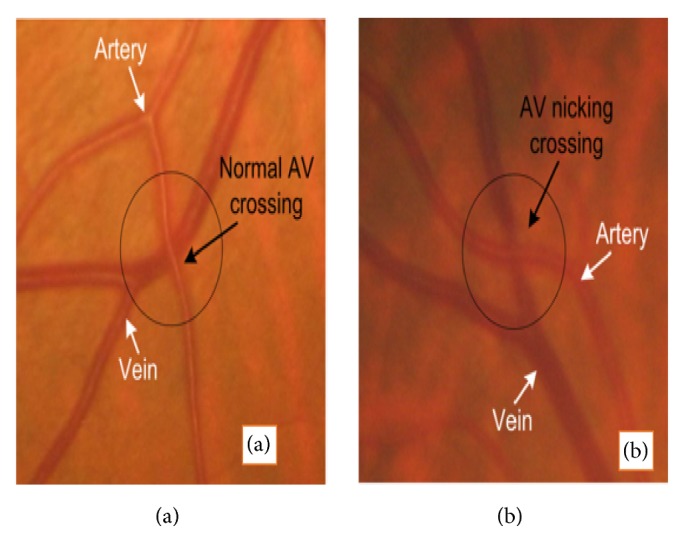
Artery-vein crossing phenomenon. (a) Common AV crossing and (b) affected AV crossing (nicking) [30].

AV nicking or AV nipping is abnormality phenomenon observed in the retinal microvascular structure during ophthalmological imaging which shows the characteristics view of crossing a small artery over a vein. Due to this arteriovenous crossing, the vein inflates either side of artery cross-section point. In several types of research related to retinal imaging, AV nicking has been found as the early indicator of eye-related diseases such as BRVO, diabetes, hypertension, and acute stroke [30, 60, 61].  Figure 2 shows the normal and affected AV crossing scenario.

The study of [30] proposed a computer-based algorithm to calculate the AV nicking severity applying the image processing techniques like image acquisition, segmentation, artery-vein classification method, and method for measuring vessel width. They validated this experiment on 47 high-resolution retinal images that were obtained from two different population-based studies. The AV nicking values obtained from this research showed a great correlation with expert grading and the accuracy of this finding is 88-89% which clarifies that severe and moderate AV nicking can be detected more precisely [30].

### 3.2. Microaneurysm Detection

**Figure 3:**
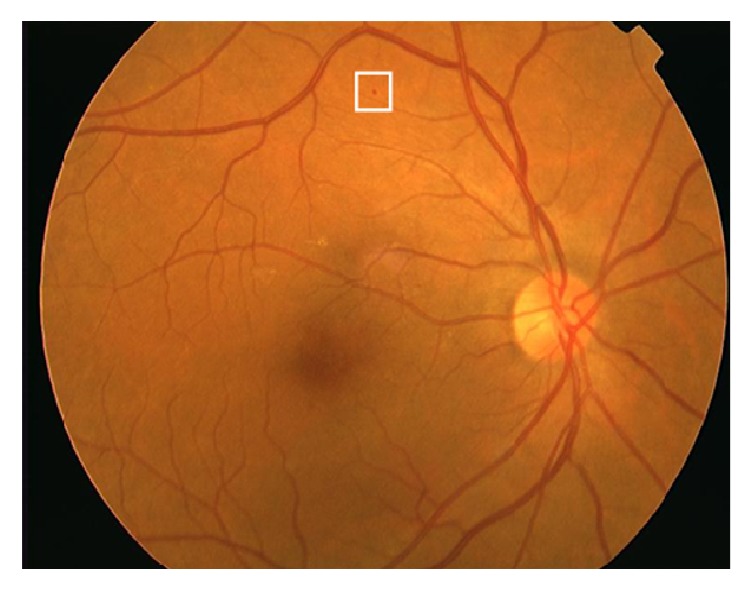
Retinal fundus image with microaneurysm (inside the white square) [31].

Generally, microaneurysm is a specific small area that is seen in the microvasculature of fundus eye image which looks like a swelled balloon. Microaneurysm is recognized as the biomarker of diabetic retinopathy as well as the presign of ischemic stroke or acute stroke that is generally observed in the fundus retinal images after analyzing the eye images [19, 48].  Figure 3 shows retinal fundus image with microaneurysm (inside the white square).

An automated microaneurysm detection method was investigated in [31] which was applied on the 50 training cases from Retinopathy Online Challenge (ROC) dataset and they came out with 45% sensitivity with their proposed method when false positive rate per image was 27. Authors of [62] used MA-Tracker, a semiautomated method, to observe the relation between the state of diabetic retinopathy and microaneurysm and they experienced that the formation rate of microaneurysm is a better bioindicator of the development of diabetic retinopathy to clinically significant macular edema (CSME) among the patients of type 2 diabetes with Nonproliferative Diabetic Retinopathy. Authors of [63] developed an automated algorithm that can count the microaneurysm and detect the turnover of microaneurysm from a digital Fluorescein angiogram repeatedly which dealt with 64 images of diabetes patient and they observed that this algorithm showed 82% sensitivity along with 2.0 false positive rate per image.

### 3.3. Cotton Wool Spot Detection

**Figure 4:**
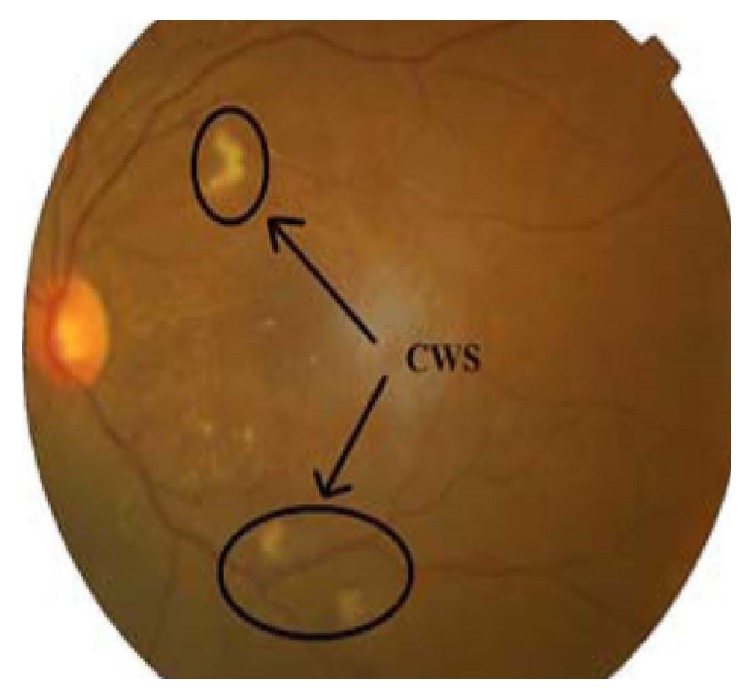
Cotton wool spot in retinal fundus image (in black circle) [32].

In the fundoscopic test of the retina of human eyes, Cotton Wool Spots (CWS) are found as the abnormality which looks like a white fluffy patch. They are also considered as the retinal infarcts which mean the damage of tissues as a result of insufficient oxygen supply because of the blockage of blood supply to the tissues and this mostly happens in the retina of the diabetic patient that leads them to be in risk of acute stroke [32]. The configuration of CWS is found similar in the fundus eye images of both diabetes and hypertension patient ([64]; Irshad & Akram, 2015;).  Figure 4 displays the CWS in fundus retinal image inside the black circle.

Researchers of [65] developed an automated system based on machine learning to detect the CWS and differentiate this from drusen existing in color images that were collected from diabetic patients and the researchers were able to obtain the sensitivity pairs of 0.95/0.86 to detect CWS.

### 3.4. Hard Exudates Detection

**Figure 5:**
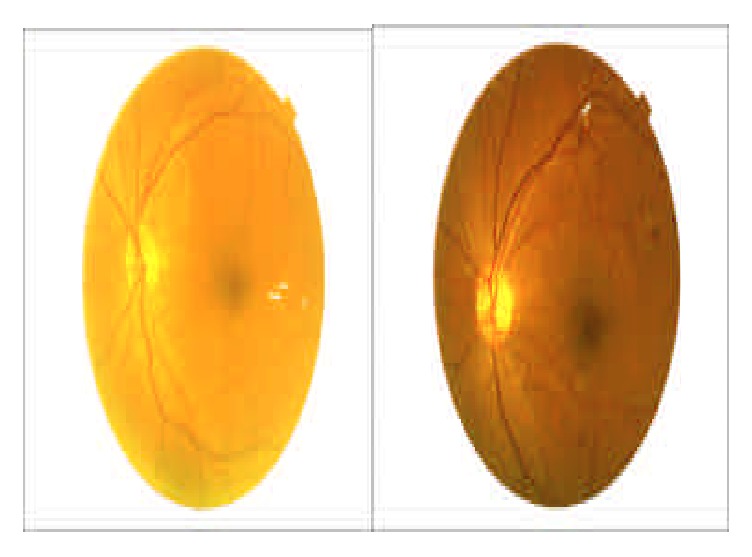
Hard exudates in fundus retinal image [33].

Hard exudates found yellow or white flecks in the outer layer of the retinal vasculature of those humans who are affected by diabetes. More generally, hard exudates are the microaneurysm that is found as white dots without blood in the lumen. Sometimes these exudates are deposited along with the vein of the retina. Hard exudates are bright interretinal protein deposition and considered as the hallmark of diabetes [66].  Figure 5 shows the hard exudate in the fundus retinal image.

An automatic image processing technique was developed by authors of [67] following Fisher's Linear Discriminant Analysis with a dataset containing 58 fundus retinal images concentrating on the variables like quality, color, and brightness which gained 88% sensitivity with the false positive mean number 4.83±4.64 per image. They used performance evaluation criterion based on the lesion and obtained 100% accuracy of image-based classification [67]. The study of [68] proposed an automated image segmentation algorithm to segment the exudates in fundus retinal images from DRIVE and STARE dataset using their green component and preprocessing steps such as contrast adjustment, average filtering, thresholding, and finally they experienced the 96.7% sensitivity [68]. The authors of [33] also performed an experiment that showed the improvement of accuracy in detecting hard exudates in retinal images and they achieved 100% and 74% specificity.

The authors of [69] detect hard exudates using the mixture model and thresholding technique to isolate the exudates from the background and then applied edge detection to differentiate exudates from CWS. This formula showed 90.2% sensitivity and 96.8% positive predictive value. The accuracy of the image-based classification was satisfactory as the 100% sensitivity and 90% specificity were obtained [69].

### 3.5. Focal Arteriolar Narrowing Measurement

**Figure 6:**
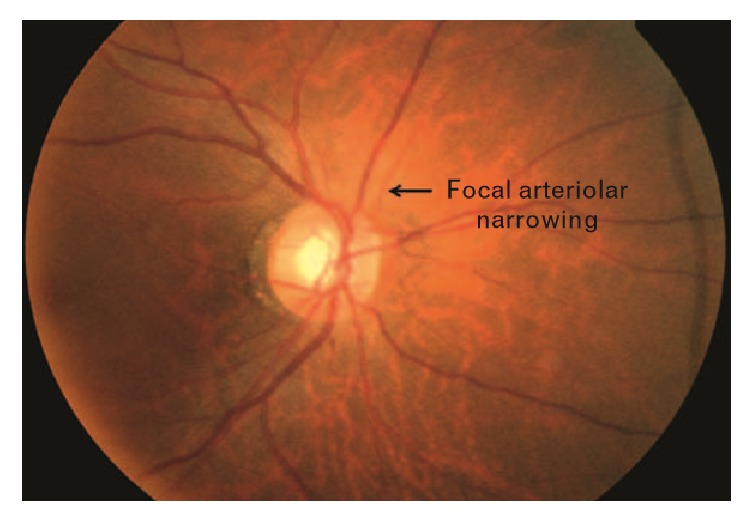
Focal arteriolar narrowing indicated by black arrow [34].

Focal arteriolar narrowing is also a symptom for the early detection of hypertension and acute stroke. Focal arteriolar narrowing generally occurs with the increasing arteriolar blood pressure from 130mm Hg to 160mm Hg that affects the arteriolar wall [70]. The study of [71] investigated the relationship between the arteriolar narrowing and hypertension and found that the hypertension increasing rate is higher among the people with arteriolar narrowing.  Figure 6 shows the focal arteriolar narrowing in fundus retinal image.

### 3.6. Vessel Width Measuring Method

As the retinal microvascular sign for predicting the stroke possibility, artery and vein diameter were widely examined to obtain more accurate measurement system and, at the very beginning of this study, AVR or arteriolar Length to Diameter Ratio (LDR) has been normalized to compute artery and vein diameter. But Atherosclerosis Risk in Communities (ARIC) and Beaver Dam Eye Study (BDES) did not find any association with AVR or LDR and stroke. However, when the measurements of retinal vessel diameter have been normalized to optic nerve head diameter association with stroke was found. Central Retinal Artery Equivalent and Central Retinal Vein Equivalent were explored to meet the challenge raised in the study of retinal vascular dimension when retinal microvascular anatomy has been found different in different individuals [72].

Retinal vessel widening is a preindication of hypertensive retinopathy and researchers found there is an association with retinal vessel diameter and acute stroke [19].  Table 1 shows the association between retinal vessel diameter and stroke.

### 3.7. Haemorrhages Detection

**Figure 7:**
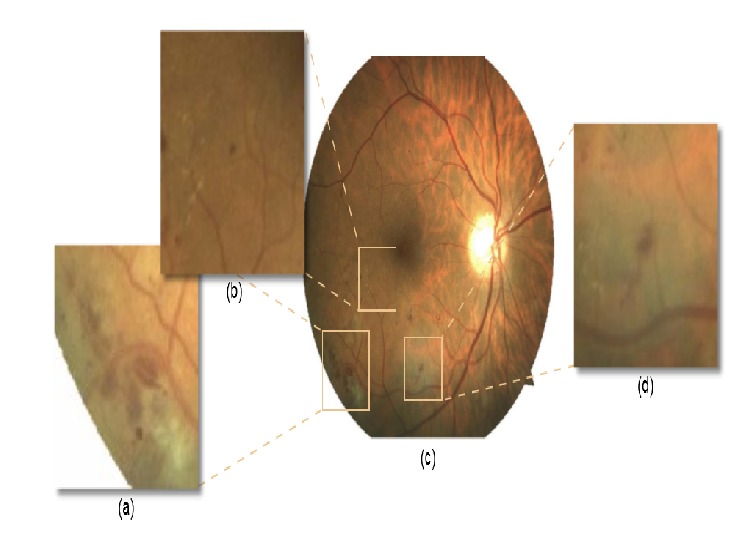
Different types of haemorrhages in fundus retinal image [35].

Haemorrhage is an abnormality that appeared in the retinal blood vessels of human eye due to the bleeding in the light-sensitive tissues on the back wall of the eye. Haemorrhages are generally seen in the retina of people with hypertension. Haemorrhages are observed in different shapes with red color and this shape can be correlated with the depth in the retina [35, 73]. The intensity of blood vessel and haemorrhages are similar and the only way to identify the haemorrhage is to eliminate the blood vessels from the blood vessels with haemorrhages which can be done by using ball-shaped Structuring Element (SE) of size 6 and size 25 simultaneously [74].  Figure 7 shows (a) large superficial haemorrhages, (b) fundus image with haemorrhages, (c) detail dot haemorrhages, and (d) bolt haemorrhages.

## 4. Generic Feature Extraction Process of Retinal Vasculature

Image processing is the method of applying mathematical operations in signal processing systems where image or video is fed as input and the output also is either image or a group of features or parameters that are related to the image [75]. Digital imaging accomplishes functions on a digital image. It is being used in image enhancement, data compression, and machine vision and deals with difficulties from edge detection to pattern recognition and reconstruction [76, 77]. In biomedical engineering, digital image processing is being applied in many researches and diagnosing the diseases, planning and supervising treatment for that disease, and monitoring the state of diseases simultaneously [78]. Digital image processing is playing an important role in medical sector to reduce the involvement of observers in avoiding unexpected errors and getting a more precise result [79].

Many life-threatening cardiovascular diseases like diabetes, hypertension, and stroke are related to the early change of the caliber of retinal microvasculature. It is the boon of modern acquisition technology that high-resolution retinal images can be captured easily for analyzing the risk of stroke among the individuals that are at risk of cardiovascular diseases and stroke. It is also responsible for enlarging the database of retinal images. The development of automated and computer-aided quantitative measurement techniques based on image processing for monitoring the changes of this microvascular caliber of the human retina has become the situation demand to ensure the maximum accuracy in detecting these destructive diseases and avoid the bulkiness of current diagnostic systems [80].

**Figure 8:**
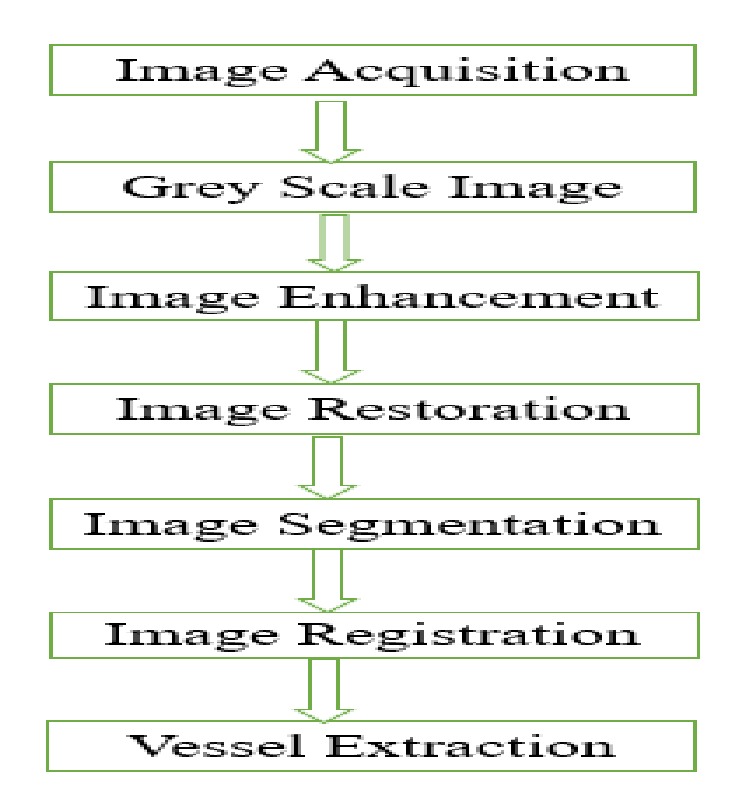
Conventional feature extraction process.

There are three major operations performed in image processing to process an image for extracting special features of the image. The first one is the enhancement of input image like contrast improvement, the second one is image restoration which means deblurring image, and the third one is the segmentation of the image, which means separating the certain portion of the image that is considered as the area of interest [81]. The related image processing techniques for extracting different features of fundus retinal image are described in Figure 8.

### 4.1. Acquisition of Fundus Retinal Image

The term image acquisition can be defined generally as obtaining an image from any hardware based source. Image acquisition is the initial and most significant step of image processing. The performance of processing image for any intended job extensively depends on the performance of image acquisition. Retinal fundus imaging can be explained as the process of obtaining a two-dimensional representation of three-dimensional semitransparent tissues of retinal microvasculature projecting on to the imaging plane and then reflecting the required amount of light [82].

A technique was developed in [83] for generating seamless, high-quality, and wide field montage which could be good for real-time photo documentation of the disc and macular abnormalities. The technique used alignment with high accuracy and blending of partially overlapped slit lamp biomicroscopic fundus image [83]. The study of [84] developed an algorithm to obtain fundus intensity image using Scanning Laser Ophthalmoscope (SLO) quality from the main spectra that were measured with spectral-domain Optical Coherence Tomography (OCT) and this algorithm provides fundus and OCT images together that can avoid the complexity of registering the fundus feature of any cross-sectional OCT image. Afterwards, this algorithm was extended to generate high contrast shadow-grams of the retinal blood vessel to facilitate the OCT data registration to the further imaging systems [84].

The study of [85] demonstrated a technique for high-speed ultrahigh-resolution 3-dimensional OCT retinal imaging with and retinal imaging protocols using Fourier domain detection. This technique used a dense raster scan pattern to obtain the three-dimensional OCT data of macula and optic disc. Retinal and interretinal layer and nerve layer thickness can also be mapped using this system [85]. The study of [86] also developed a real-time imaging approach using a single pixel camera for capturing a perfect fundus retinal image.

In modern ophthalmology, there are a lot of image acquisition techniques already developed even using the smartphone. The built-in camera technology, exciting development of cloud storage, and technology for accessing electromedical history using smartphone have encouraged the physicians to use the smartphone in ophthalmic imaging and lead to a dream to have a teleophthalmology system fully based on a smartphone. Welch Allyn first developed an imaging adaptor based on smartphone attaching an iPhone with Welch Allyn Panoptic Ophthalmoscope that can take pictures of retina including the iExaminer App [87]. The noble design of a compact, slim, smooth, and 3D printed attachment that allows high-quality fundus image coupling smartphone to indirect ophthalmoscopy condensing lenses was reported in [87].

### 4.2. Grey-Scale Retinal Image Processing

The grey-scale image can be represented as data matrix and the value of this matrix illustrates the shades of grey. If the elements of a grey-scale image are of class “unit8,” the range of their integer values will be [0, 255] and if the elements are of class “unit16” then the range will be [0,65535]. When the class of a grey-scale image is “single” or “double,” normally its value can be scaled in the range [0,1] [88].

Converting an RGB color image into the grey-scale image is the first step of many image analysis workflows because in the grey-scale image the amount of information is simplified and maintained only the information related to the features of the image like edges, regions, blobs, and junctions that are needed to be analyzed [89]. The following transformation equation is used to convert the RGB color image into grey-scale image:

(1) Igrayn,m=αIcolorn,m,r+βIcolorn,m,g+γIcolorn,m,b89.

Here I_gray_ is grey-scale image, I_color_ is color image, *α*= 0.2989, *β*=0.5870, *γ*=0.1140, (n,m) is pixel location with grey-scale image, and (n,m,c) is a channel at pixel location (n,m) in the color image for channel c in red r, blue b, and green image channel [89].

**Figure 9:**
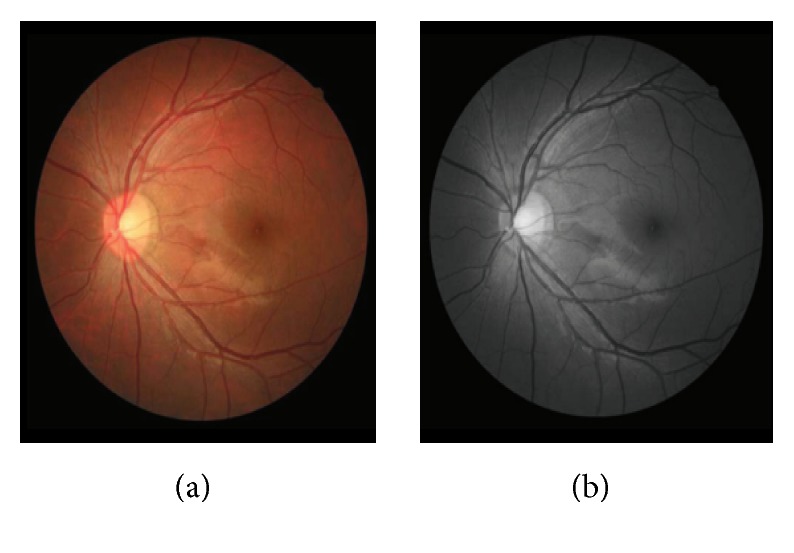
Original image (a) and grey-scale image (b) of the retina [36].

There is the possibility of losing the important features of an image like structure, sharpness, contrast, and shadow during the conversion of the color image into grey-scale image. The study of [90] proposed an algorithm for converting the RGB image into the grey-scale image which can do RGB approximation, reduction, and addition of luminance and chrominance and sustain the structure, sharpness, contrast, and shadow of the original RGB color image in the resultant image. The study of [91] presented a formula based on the Singular Value Decomposition (SVD) to measure the graphical and scaler values of distortion caused by the noise sources. As a step of fundus retinal image processing, the raw RGB color image is converted into grey-scale using the green channel because the blood vessels of retinal microvasculature appear more contrasted through green channel [36].  Figure 9 shows the original image (a) and grey-scale image (b) after conversion.

### 4.3. Enhancement of Fundus Retinal Image

Image enhancement is the procedure to synthesize the digital images for making the output more acceptable to display or for further analysis of that image. Image enhancement is also performed to adjust the contrast and normalize the images. Most often the fundus retinal images do not illuminate uniformly and show local luminosity and irregularity in contrast [92]. The fundus retinal images of human have different outlook due to the skin pigmentation of the subject. To gain a good sensitivity and specificity in detecting lesion of fundus retinal images following any automated analytical method, color normalization of that image is a must [93].

Some of the popular image enhancement techniques are removing the noise from the image, sharpening the edges of the image, blurring the image, and so on which can be accomplished by spatial domain filtering because spatial domain filtering can directly act on the images and change the pixel values of the image through some specific procedure [89].

The authors of [93] demonstrated a system of color normalization performing intraimage shade-correction interimage histogram normalization and they detected the microaneurysm in fundus retinal images applying the effect of their technique. The authors of [92] also proposed a method for normalizing the contrast and luminosity in both intra- and interimages of the human retina and the researchers came out with the average luminosity irregularity reduction of 19% covering maximum 45% and an average improvement of contrast of 34% covering maximum 85%.

#### 4.3.1. Retinal Image Restoration

Image restoration can be defined as recovering or reconstructing a degraded image utilizing the earlier concept of image degradation phenomenon. The techniques of restoring an image are designed towards modeling the degradation and using the reverse procedures to regain the real image.

In fundus retinal image analysis restoration process plays a vital role to avoid the blurring and uneven illumination due to the image acquisition process. The authors of [47] proposed a technique for the restoration of the color retinal image through multichannel blind deconvolution. Basically, this technique is designed by composing image registration, uneven illumination compensation, and segmentation that had been validated by applying on both synthetic and original retinal image. It was experienced that this method is able to restore the degraded retinal image and can detect and picturize the structural changes of the retinal image too [47].

Fundus eye image can be degraded with blur due to the inappropriate acquisition or congenital optical shedding in the eye. The restoration process is either space-invariant or space-variant and maximum existing algorithm for image deblurring can deal with the space-invariant blur but cannot work with space-variant (SV) blur image [37]. The authors of [37, 77] presented a retinal image restoring algorithm that can deal with both unknown and space-variant blur and formulate this algorithm and they shaped the blur, interpreting linear operation for convolution with Point-Spread Function (PSF) that changes with the position in the image.

**Figure 10:**
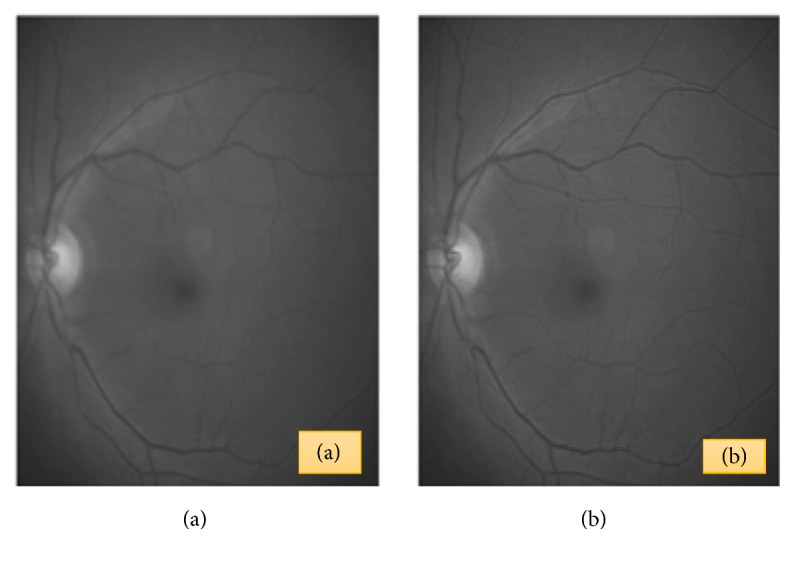
(a) Original and (b) restored image using Space-Variant Point-Spread Function [37].

The study of [94] also presented a method to obtain a true estimation of Point-Spread Function (PSF) for restoring retinal image through space-invariant or space-invariant blind deconvolution based on the decomposition in Zernike coefficient of the estimated PFSs to determine the actual PSFs.  Figure 10 shows the original and restored fundus retinal image.

The study of [95] designed a method to recover the spectral retinal image of the common RGB image using fuzzy c-means clustering for quantizing the data of image and radial function network for learning the mapping from RGB representation to the spectral space. And authors of [95] used a spectral quality metric to evaluate their experiment result compared with a set of retinal images having both spectral and RGB image and found the accuracy of their output relatively high.

### 4.4. Segmentation of Fundus Retinal Image

Image segmentation means the process of dividing an image into its specific constituent Regions of Object of Interest (ROI) to make that image more significant and smooth to analyze. Segmentation of an image can be done for isolating wide ROI and once the ROI is achieved segmentation process can be stopped.

**Figure 11:**
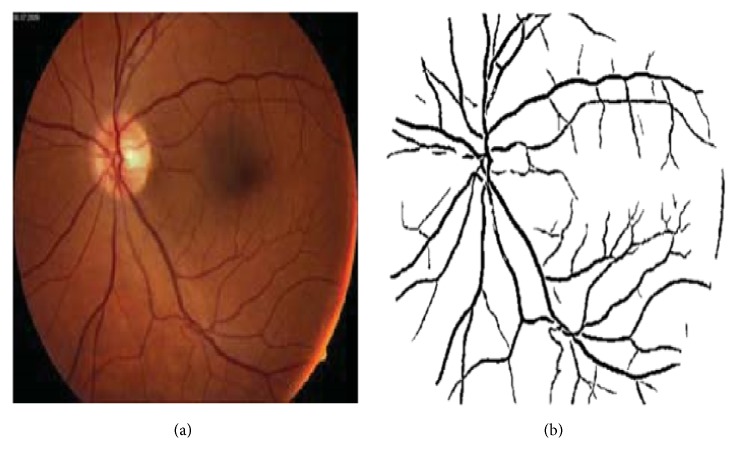
(a) Fundus retinal image, (b) original and segmented [38].

Extraction of different properties of fundus retinal image is either qualitatively or quantitatively. The extracted features have become the key fact for diagnosing many severe cardiovascular diseases. As the changes of different features of retinal microvasculature such as vessel tortuosity, branching coefficient, branching angle, vessel widening or narrowing, arteriovenous nicking, existence of hard exudates, CWS, microaneurysm, and haemorrhages have been detected as early signs of many cardiovascular diseases leading to stroke [1, 3, 5–9, 19, 49, 60, 61], segmentation of fundus retinal image is a must to detect and measure the abnormalities of retinal microvasculature. There are a lot of segmentation procedures based on image modalities, automation or semiautomation, application domain, and some other different factors. Image segmentation algorithms and techniques can be categorized into six main sections such as rigid based vessel segmentation, parallel multiscale feature extraction, and region growing, artificial intelligence based methods, hybrid filtering, miscellaneous tube-like object detection methods, and neural network (NN) based methods [96].  Figure 11 shows the original fundus retinal image of a left eye with normal blood vessel network and segmented blood vessel image.

### 4.5. Edge Detection for Segmentation

Edge detection is one of the particular procedures of image segmentation to extract boundaries, discontinuities in the intensity of an image that can be used for analyzing that image because edges convey important clues for finding out the key fact of interest. Some structural information like the boundary of the object, illumination, geometry, and reflectance can be obtained analyzing the detected edge [97].

There are several edge detectors based on the principle of function edge that performs better in images with less noise. The available edge detectors based on the function edge are Sobel Edge Detector, Prewitt Edge Detector, Roberts Edge Detector, Laplacian of Gaussian (LoG) Edge Detector, Zero Crossing Edge Detector, and Canny Edge Detector (Gonzalez, Woods, & Eddins, 2009).

Edge detection is an important way to extract the key features of retinal microvasculature. A variety of edge detecting algorithms are being developed in fundus retinal image processing. The authors of [98] developed a fundus coordinate system combining region growing and edge detection to detect the exudates, an important feature of the retinal image, and modified active shape model to detect the disc boundary of fundus retinal image. The success rate of their algorithm in terms of detecting optic disc boundary is 94% and the sensitivity and specificity of detecting exudates are 100% and 71%, respectively [98].

A template-based retinal image segmentation was presented in [99] in which morphological and edge detection technique employing the circular Hough transform for approximating the circular optic disc boundary was applied.

### 4.6. Image Thresholding

Image thresholding technique is basically an image segmentation technique that is applied to alter a grey-scale image into the binary image to differentiate the objective point of interest. Thresholding is most suitable for analyzing the images with high contrast.

Thresholding is an important technique that is widely used for the segmentation of fundus retinal images to analyze the microvasculature. Thresholding is used in retinal image processing only to highlight the features of interest and avoid the features that are not important for analyzing the image. The authors of [100] used entropy-based thresholding as subtechnique to keep the spatial structure of vascular tree segments in their retinal image analysis algorithm to detect and extract the blood vessels. A knowledge-guided adaptive local thresholding algorithm based on the verification-based multithresholding probing scheme was proposed in [101] to detect the blood vessel in fundus retinal image.

### 4.7. Image Registration

Image registration is an image processing technique designed for aligning multiple images of the same configuration. Generally, image registration is the procedure to transform multiple datasets, photographs, and times depth into one coordinate system to accomplish several tasks like image rotating, scaling, and skewing. The image configuration of the same object can vary due to the acquisition at different times, different acquisition devices or acquisition inefficiency caused by the variation of camera angle, movement of the objects, orientation, sensor resolution, distance, including other crucial facts and the image registration process; organized to perform the alignment of the geometrical aberrated images, based on a standard image [88].

Image registration plays a supreme act in analyzing the fundus retinal image because it is the way to protect and provide the accurate information that is critically important for diagnosing the related diseases. The study of [102] proposed an algorithm applying a nonlinear registration method based on correlation tracking to enhance the retinal imaging with a high spatial resolution for clear and precise detection of retinal abnormalities. The local correlation was also analyzed in [102] experiment to observe the actual movement of an image in a different time period like the variation in optical flows that can be the feature of interest of diseases diagnosing.

**Figure 12:**
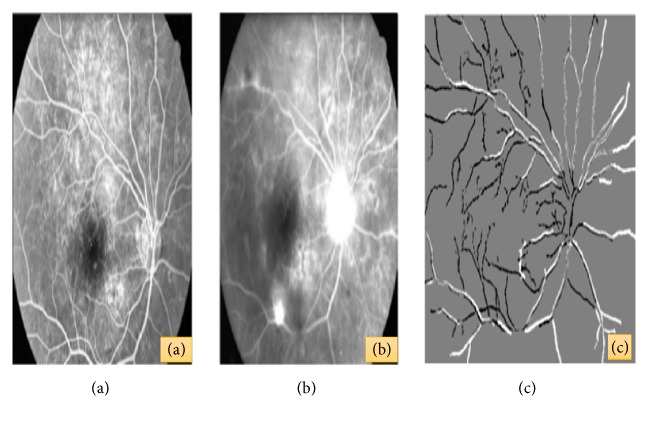
(a) Original fluorescein image. (b) Fluorescein image two years later of temporal registration. (c) Final result of the registration [39].

A hybrid retinal image registration technique for Early Treatment Diabetic Retinopathy Studies (ETDRS) was designed in [103]. This technique was able to extract retinal microvascular structure applying local entropy-based thresholding and maximized the mutual information of binary image pair to estimate the zeroth order translation. Image quality was assessed regarding the definition of ETDS based on the translation model and then finally affine/quadratic model estimation had been applied after image pair was accepted [103]. The necessity of temporal image registration is to observe the different steps of disease and the detection of lesions in fundus retinal image can be improved by multimodal image registration. The study of [39] presented a temporal and multimodal retinal image registration technique based on point correspondence. This method first detects the vascular tree and labeled the bifurcation point. After that, it matches the probability for matching two points computing an angle-based invariant and uses a Bayesian Hough transform to distinguish the respective similarities and finally it computes a fine estimation to choose the best similar transformation for registration [39].  Figure 12 shows (a) original fluorescein image; (b) fluorescein image two years later of temporal registration; (c) final result of the registration [39].

An advanced sequential processing method for the retinal image was proposed by authors of [104] where they used cross-correlation followed by a fine registration employing parabolic interpolation on the peak of the cross-correlation, maximum-likelihood estimation for precise registration, and a combination of peak tracking and Procrustes transformation to measure angle rotation of the fundus retinal image.

### 4.8. Vessel Extraction of Fundus Retinal Image

Extracting the vessel tree of retinal microvasculature is an important step to analyze the microcirculation for the detection of retinal diseases. The study of [105] reported a retinal blood vessel segmentation algorithm following the scale-space analysis of the first and second derivative of the intensity image. A parallel multiscale feature extraction and region growing algorithm based on ITK (Insight Segmentation and Registration Toolkit) was developed for retinal blood vessel segmentation and showed that this method is effective for high-resolution retinal image analysis [80]. The study of [106] designed an automated segmentation and reconstruction method for 3-dimensional retinal vessel tree extraction, vessel detection, and vessel calibers estimation assembling the near-infrared reflectance retinography information with OCT section.

A postprocessing model for extracting features of fundus retinal image was introduced in [107–109] that segment the retinal microvasculature to extract the blood vessel applying Krisch Edge Detector and identify the true vessel using Graph Tracer. An automated segmentation method was developed in [110] for segmenting the image of retinal microvascular structure to identify the true vessel using pixel's feature vectors that are the combination of pixel's intensity and continuous two-dimensional Morelet Wavelet transform response adopted at multiple scales.

The study of [111] proposed a vessel segmentation technique using nonlinear diffusion filter for the smoothening vessel to their principal direction, compound vessel enhancement filter combining eigenvalues of the Hessian matrix, matched filter response, and edge constraints of multiple scales for vessel enhancement and then multiple thresholding was applied for centerline tracking. The study of [112] introduced an automatic system to enhance and segment true blood vessel of fundus retinal image using 2-dimensional Gabor wavelet and multilayered thresholding, respectively.

The authors of [113, 114] combined several techniques for the segmentation of fundus retinal image that can detect the vessel centerline and slice the morphological bit plane to extract the vessel tree of the human retina. The study of [38] presented two different methods for retinal blood vessel segmentation where the first method is the procedure for region growing using the hysteresis thresholding which later applied to the response vector similarities of adjacent pixels within the fundus image and the second method was developed based on region growing and directional response vector similarities. For the latter method response vector was calculated through template matching with general Gabor function [38].

The study of [115] proposed an algorithm for vessel segmentation and vascular network extraction based on the multiscale line-tracking [116] procedure where map quantization of the multiscale confident matrix was applied to generate the initial vessel network and then disconnected vessel line was restored and noisy line eliminated applying the median filtering to the generated vascular network and finally directional attributes of vessel and morphological reconstruction was applied as postprocessing for avoiding the faulty areas. An automatic blood vessel extraction technique was presented in [117] in which curvelet-based contrast enhancement, match filtering, curvelet-based edge extraction, and length filtering were used to extract the blood vessel.

The study of [118] proposed a vessel segmentation method to detect the blood vessel in the retinal image based on a probabilistic tracking method. The study of [119] presented a multiconcavity-based segmentation model that deals with the lesion in retinal microvasculature. There were four different techniques demonstrated in [120] for blood vessel segmentation based on Edge Enhancement Edge Detection, Image-Line Cross-Section, Continuation Algorithm, and Modified Matched Filtering that can work with abnormal retinal images having exudates, drusen, and low vessel contrast. A supervised method for detecting blood vessel in fundus retinal images was designed in [121] based on a blueprint of NN for pixel classification and a 7D vector computation that is composed of moment-invariant based features for pixel representation and grey-level. The study of [98] examined a novel method for feature extraction of color retinal image applying Principal Component Analysis, Active Shape Model, fundus coordinates system, and a combined region growing and edge detection technique to locate optic disc, detect the shape of an optic disc, describe the features, and detect the exudates in color retinal image, respectively.

The blood vessels in a fundus retinal image that appear in a higher illumination variance area are found missing if the background removal method for segmentation is applied because the background and intensity values of the blood vessel are almost the same. The study of [122] presented a robust method for blood vessel segmentation to change the illumination intensity applying background estimation that was calculated by a weighted surface fitting method with a higher degree polynomial.

#### 4.8.1. Automatic Identification of Vessels

Identification of retinal artery and vein in fundus retinal images is one of the most important stages for detecting the changes of either qualitative or quantitative features in retinal microvasculature and further extraction of the respective features that have association with risk cardiovascular diseases. To ensure the effectiveness of the developed systems for clinical diagnosis of diabetic and hypertensive retinopathy, the vessel identification must be accurate. The authors of [123] designed an automated method for the artery and vein identification in dual-wavelength, 570nm and 600nm, retinal images. In this system they utilized the structural feature, relative strength of the vessel central reflex, and the ratio of the vessel optical densities from images at oxygen-sensitive and oxygen-insensitive wavelengths as the functional feature for each vessel segment to differentiate the artery from vein. The study of [123] employed the dual-Gaussian model in which parameter was estimated using a robust M-estimator to compute the relative strength of the central reflex. For the identification of vessel type whether it is artery or vein, the structural and functional features were combined in four classifiers and it was observed that the Support Vector Machine gave the best result with 97% and 90% positive rates for bot arteries and veins, respectively [123].

The study of [124] developed an automatic technique to identify the artery and vein in illumination-corrected retinal images combining Gaussian Mixture Model, Expectation-Maximization unsupervised classifier, and a quadrant-pairwise approach. The obtained specificity and precision of this technique were 0.8978 and 0.9045 for artery, respectively, and 0.9591 and 0.9408 for vein, respectively. The authors of [125] designed a vessel classification system employing the linear discriminant classifier in their proposed method for the measurement of AVR which gave the 92.8% accurate classification results. An automated system for the artery and vein classification was proposed in [126] that provides the classification result for the vasculature analyzing the intersection points (graph nodes) and assigning one of two labels to each vessel segment using graph links. The graph-based labeling results were combined with an intensity features set to perform the final classification and that showed the 89.8%, 8834%, and 87.4% accuracy for the images of VICAVR, INSPIREVR, and DRIVE database, respectively [126].

## 5. Image Processing-Based Blood Vessel Diameter Measurement for Stroke Risk Detection

Vessel diameter was measured manually by selecting a region of 512 × 512 pixels of a digital image and then calculating the linear distance between two points of opposite edges of a vessel of a digital image. The Gaussian measurement of vessel diameter was performed by analyzing a set of vessel characteristic parameters. These parameters were determined by fitting a double Gaussian model to the intensity cross-section of the vessel. The function below illustrates the intensity cross-section of the vessel and a modified Levenberg-Marquardt [127] least squares method was used to determine the parameters a1 to a5 and a7. The calculated vessel width would be 2.33a3 [128].

(2) $$\begin{array}{c} I\left(\;x\;\right)=a1e^{-{\left(\left(x-a2\right)/a3\right)}^{2}}+a4-a5e^{-{\left(\left(x-a2\right)/a7\right)}^{2}}\left[\;127\;\right]. \end{array}$$

Sobel edge detection masks were applied to perform Sobel measurements identification of vessel edge position that determines the vessel edge position from the average position of maximum edge strength which was averaged over a three-pixel window. Linear regression within sliding window filter was used to create Sliding Linear Regression Filter (SLRF) measurement identification of edge positions. The SLRF were used to measure the vessel diameter and it was seen that the performance of SLRF was greater than the manual measurement system.

**Figure 13:**
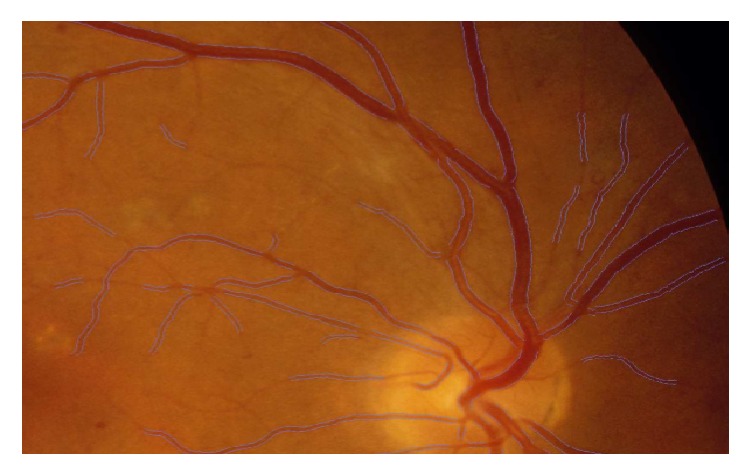
Widened blood vessel of retina [23].

Several studies revealed that there was no association with an arteriolar width decreasing and incident stroke and prevalent stroke rather venular widening was associated with incident stroke [18, 129]. AVR was also analyzed to determine the relation between AVR and incident stroke and prevalent stroke as the arteriolar and venular widening is responsible for changing the value of AVR. No association was found between incident stroke and AVR but the association between AVR and prevalent stroke was examined [18].  Figure 13 shows the widened vessel of retinal vasculature.

The researches of [130] developed an algorithm for measuring the diameter of the retinal blood vessel to subpixel accuracy by applying the two-dimensional difference of Gaussian model. The researchers came out with 30% more precision in comparison with Zhou's Gaussian model, Brinchmann-Hansen's half height and Gregson's rectangular profile and accuracy of a third of a pixel [130].

A semiautomatic vessel width measuring method named Computer-Aided Image Analysis of Retina (CAIAR) was formulated in [131], in which computer-generated lines, similar to the blood vessel with prefixed frequency, amplitude, and width were used. The resultant width values of retinal blood vessel obtained from this formula were found as less correlated with the ophthalmologist grading [131]. The study of [23] proposed a graph-theoretic algorithm for measuring the vessel width. The work in [20] measures the blood vessel diameter based on thresholding segmentation and training step determining the characteristic point using Douglas-Peucker algorithm. The work in [20] detected vessel contour using active contour and measured vessel diameter using Heron's formula.

An algorithm based on the Graph Tracer method was developed in [132], which can identify the true blood vessels, appropriate bifurcations, and crossover. Multiscale Line Tracing was applied for segmentation and blood vessel annotation tool was used to measure the width of the identified blood vessel and this technique achieved 94.6% accuracy in measuring the diameter of the blood vessel. The study of [133] proposed an algorithm for measuring vessel diameter based on intensity profiles and Dijkstra's shortest path algorithm. The work of [130] also presented a vessel diameter calculating method based on 2D modeling that is more precise than HHFM and Gregson algorithm.

The study of [24] introduced a graph-based algorithm to measure the width of the retinal vessel that segmented both vessel edges following a two-slice, 3D surface segmentation problem model which was converted into a minimum closed set in a node-weighted graph problem in the next step. In their experiment it was shown that more accurate measurement of the vessel width of fundus images can be obtained with larger standard deviation (*σ*) and the success rates of this algorithm on four datasets, KPIS, CLRIS, VDIS, and HRIS, of REVIEW database are 99.4%, 94.1%, 96%, and 100%, respectively [24]. The authors of [25] proposed a vessel diameter measurement algorithm based on Gaussian modeling combining a series of second-order and higher order Gaussians to design the vessel profile and they used the sigma parameter of generalized Gaussians to the vessel boundaries. The accuracy and precision of this method for CLRIS are −1.574 and 1.691 and for VDIS are −0.443 and 1.182. They compared their result with twin-Gaussian, SLRF, and manual measurement and it was claimed that the accuracy and precision of twin-Gaussian are the least [25].

The authors of [26] designed a hypothesis based method for retinal vessel width measurement applying the theory that Higuchi's dimension of the cross-section is proportional to the vessel diameter. They used REVIEW database to validate their proposed method and came out with the precision and success rate of 0.65 and 99.45%, 1.56 and 98%, 0.45 and 100%, and 1.14 and 97.8% for HRIS, CLRIS, KPIS, and VDIS datasets, respectively. The advantage of this technique is that the results cannot be degraded by edge detection performance and segmentation process as it does not depends on segmentation process [26]. The study of [21] shaped an active contour model named Extraction of Segments Profile (ESP) to measure the retinal blood vessel diameter. Though their algorithm may fail sometimes, they obtained the success rates 99.7%, 99.6%, 93%, and 100% for HRIS, VDIS, CLRIS, and KPIS datasets, respectively, of REVIEW database [21].

An automated method for vessel diameter measurement based on LDA was developed in [53] unsupervised method, which can measure the vessel diameter to subpixel accuracy for all datasets. The study of [52] proposed an algorithm for measuring the retinal vessels widths based on deformable models and which was integrated into an AVR computing framework. This method was robust against different grey color spaces.

The authors of [54] used a novel parametric surface model of the cross-sectional intensities of vessel and combination of bagged decision trees in their algorithm to estimate the retinal vessel width in fundus images. This algorithm was compared with several algorithms such as 1D Gaussian, 2D Gaussian, Gregson, HHFW, Extraction of Segment Profiles (ESP) of Al-Diri, Unsupervised Linear Discriminant Analysis based algorithm of Kumar et al., and graph-based algorithm of Xu et al. and came out with good stability, 100% success rate on all four datasets of REVIEW database [54].  Table 2 displays the performance in terms of accuracy and the applied method to shape the most recent algorithm for measuring the diameter of retinal blood vessels

In Table 2, the most widely used performance measurement of their proposed algorithm is success rate. The success rate can be defined as the ratio of the number of successful runs and the total number of runs. It is a measure of the stability of measurement that was returned [21], along with the mean and standard deviation of measurements and differences. From Table 2 it is seen that the REVIEW database was used in most of the researches, whereas the success rate of the experiment using KPIS datasets achieved 100% with the better standard deviation in maximum cases. This is because the images of this dataset were taken from the clean, large vessel segments and downsampled such as HRIS [21]. Among the applied methods described here, the [54]'s algorithm based on supervised learning performed by bagged decision trees and an extended multiresolution Hermite model showed the 100% success rate, maximum stability with a good standard deviation of the point to point difference for all the four datasets of REVIEW database. The graph-theoretic method of [23, 24], ESP algorithm of [21], Adaptive Higuchi's Dimension based algorithm of [26], ULDM of [53], and deformable model-based algorithm of [52] exhibited poor accuracy as the mean diameter of retinal blood vessels deviated largely for the CLRIS and VDIS datasets.

The success rate of [53] on HRIS dataset was degraded due to the presence of diabetic abnormalities nearby the vessel boundaries. As the images of VDIS dataset are noisy with lower resolution which is mostly used in pathological purpose, the testing of several algorithms on this dataset led to large deviation and poor accuracy result [53]. The edges of the vessels of CLRIS dataset are highly blurred which can also affect the performance of algorithm [21].

## 6. Challenges and Future Work

Fundus retinal image processing has become one of the most interesting technologies in diagnosing many cardiovascular diseases such as stroke. According to the medical study, some of the key features of retinal microvasculature convey the symptom of stroke such as the existence of hard exudates, microaneurysm, CWS, and changes in the vessel diameter. To be cooperative with the physicians in terms of detecting the early signs of this lethal condition, researchers from biomedical engineering discipline are being involved more enthusiastically. Numerous quantitative methods for quantifying the abnormal changes in vessel diameter have been developed to provide more precision in medical diagnosing. But, still, there is a scarcity of automatic vascular caliber quantifying methods with more accuracy especially when dealing with images with abnormality. The existing image acquisition technique still has limitations on the basis of automation though there are some ultramodern image acquisition techniques that have been developed which are applicable in the smartphone and the misery in autoalignment of the captured image based on a standard preregistered image. One of the potential ways to avoid this penury is to apply the image registration technique prior to image acquisition or segmentation. Images can be degraded with blur at the time of acquisition and it is either space-variant or space-invariant. Maximum image acquisition algorithm is not effective in a space-invariant blur. It is known that spectral image is more informative for retinal images. Lack of spectral retinal image is also considered as an obstacle of image processing [95].

Edge detection is also important in the workflow of measuring blood vessel diameter as several vessel diameter measuring algorithms go through this technique. Though a lot of edge detection algorithms are available, sometimes the performance of the existing algorithms degraded due to the poor local contrast and wrong illustration of the central light reflection in fundus images [97]. This scenario greatly affects the results of computational technique to measure the vessel diameter especially the smaller vessel diameter measurement. Another worst condition arises in detecting edges and measuring the vessel width if false positive or vessel discontinuities occurred [23]. To avoid this situation edge detecting technology should be developed to a great extent.

Some challenges for image registration technique as mentioned by ETDRS are the feature-based method problems in superfluous landmark points due to small overlaps between adjacent fields. The area-based technique cannot perform well because of irregular intensity distribution from defective data acquisition. Also, both feature and area-based technique can result in less accuracy due to the high-resolution images containing huge homogeneous textureless regions [103].

It is observed that graph-theoretic method has a large contribution in the development of the vessel boundary segmentation and vessel width measurement technique. Some drawback of the vessel width measurement algorithm based on the graph is unable to define the normal direction of the blood vessel that leads them to result in poor accuracy [23]. As the vessel width measurement algorithm depends on the segmentation, the inefficient segmentation method also distorts the outcome of the system.

Another most important issue is that the image quality of the databases which are being used widely to validate the designed algorithm for retinal vessel width measurement. In some cases, it was found that the performance of vessel width measurement method was affected due to the different features and qualities of the images from different datasets. Supposing the diabetic abnormalities are present in the images of HRIS dataset, highly blurred vessel edges of the image of CLRIS dataset and the noisy, pathological images with lower resolution images of the VDIS dataset are found as responsible for the degradation of the performance of the proposed algorithm. For the better accuracy and to create supreme enthusiasm among the researchers the quality of the contents of the testing image sets needs to be updated. Employment of robust retinal blood vessel segmentation algorithm also overcomes the issue of image quality. The proposed method of [134] had shown its effectiveness on the unhealthy dataset. Thus it is believed that the method is meaningful when applying on the abnormal retinal dataset.

With the rapid development of technology, retinal image processing is also marching forward with lots of novel achievements to provide the human beings with a more secured life creating a notable application that can detect the preindication of stroke with more accuracy. The latest technology of retinal image analysis is only for the image acquisition using a smartphone and that is also dependent on a large screen and power consuming devices for further processing. A fully automated image processing technique to extract the features and measure the vessel diameter of the human retinal vasculature with maximum accuracy needs to be developed. The system can be operated by using a smartphone to facilitate the regarding ophthalmologist with the most feasible and expected teleophthalmic system for the diagnosis, supervision, and monitoring diseases state of life-threatening microvascular disease like stroke.

## 7. Conclusion

The field of digital image processing has a wide variety of concerns of numerous applications. These workable applications of image processing are being used in the largest platform, medical diagnostic system. Fundus retinal image processing is one of the most fertile disciplines of digital image processing that is continuously creating a lot of robust applications with numerous novel features to facilitate the biomedical engineering sector in terms of diagnosing the diseases, planning and supervising the treatment of the diseases, and monitoring the condition of diseases together. The cause of increasing appeal of this widely ranged area of research is that the digital fundus image of human retina can be analyzed noninvasively in vivo and the objects of interest can be visualized with more accuracy.

A lot of life-threatening diseases such as stroke can be diagnosed in their early state analyzing the microvascular structure of human fundus eye image. Some of the features of retinal microvasculature such as hard exudates, haemorrhages, AV nicking, CWS, focal arteriolar narrowing and vessel width change remarkably due to that dangerous disease. Researchers are being involved in this potential field, retinal image processing, to facilitate biomedical engineering sector by developing novel techniques. These techniques can be used to extract and analyze the features of interest with more precision.

Retinal image acquisition is the initial step of retinal image processing to analyze and extract the relevant features. After the acquisition of the retinal image, it goes through several steps such as image enhancement, image restoration, image construction, and image segmentation. These processes are used to figure out the particular objects of interest and some further processing to find out the more specific features and their characteristics. There are a good number of automatic qualitative and quantitative applications that have been embodied based on these image processing techniques. These techniques can detect abnormalities in retinal vasculature such as AV nicking, hard exudates, CWS, and haemorrhages. These techniques also can measure the unexpected changes of vascular caliber such as widening the vessel diameter, which occurred in retinal microvasculature which are directly related to stroke even though the blood pressure and other relevant vascular risk factors are at the tolerable stage. Linear Discriminant Analysis, image gradient segmentation technique (ARG) for vessel segmentation, mask creation, Sobel edge detection, Gaussian Measurement, Sliding Linear Regression Filter (SLRF), and Computer-Aided Image Analysis of Retina (CAIAR) are some of the systems to measure the retinal blood vessel diameter that has been developed using image processing principle. Exploring the advancement of retinal image processing, it can be expected to have a great revolution in modern ophthalmology as this discipline depends on the screened information at a large extent.

## Figures and Tables

**Table 1:** Association between retinal vessel diameter and stroke [19].

Outcome	Study	Total Sample size	Methodology	Association with Retinal Vessel Diameter
Prevalent stroke	CHS [6]	2050	Clinical	0
	CHS [40]	1717	MRI	AVR
	ARIC [28]	1684	MRI	AVR^*∗*^

Prevalent WML	ARIC [47]	1684	MRI	0
	CHS [40]	1717	MRI	AVR
	Rotterdam [48]	490	MRI	Venular diameter

Progression WML				
3.3-year	Rotterdam [48]	490	MRI	Venular diameter
5-year	CHS [40]	1717	MRI	AVR

Incident stroke				
3.3-year	Rotterdam [48]	490	MRI	0*ƚ*
3.5-year	ARIC [5]	10 358	Clinical	0
5-year	CHS [40]	1717	MRI	AVR
5-year	CHS [49]	1992	Clinical	Venular diameter
7-year	BMES [8]	3654	CT or MRI	0#
8.5-year	Rotterdam [50]	5540	CT or MRI	Venular diameter
10–12 years	Pooled BDES BMES [51]	7494	Clinical	0

Stroke mortality			ANDI	
7-year	BMES [8]	3654	Death certificate	0
10-year	BDES [24]	413 cases		AVR
		1251 controls	Death	
			certificate	
10–12	Pooled BDES BMES [51]	7494	or ANDI	0

**Table 2:** Accuracy and applied method of a recently proposed algorithm for measuring the vessel diameter [20, 21, 23–26, 52–54].

No	Paper Info (Authors)	Contribution	Feature	Method	Database REVIEW	Results				
						Accuracy				
						Success rate%	Measurement (in pixels)		Difference (in pixels)	
							*µ*	*σ*	*µ*	*σ*
1	[23]	Algorithm for retinal vessel boundary detection and width measurement	Retinal blood vessel width	Graph-Theoretic method	HRIS 90 segments 2368 vessel profile	100	4.54	1.23	0.18	0.47
					VDIS, 79 segments, 2249 vessel profile	96.0	8.59	2.44	−0.29	1.13

2	[52]	A method for measuring the retinal vessels widths and computing AVR in REVIEW database.	Retinal blood vessel width	This algorithm is based on deformable models and integrated into an AVR computing framework	REVIEW 5066 vessel profiles					
					KPIS, SIRIUS					
					G	100	6.20	0.63	−1.28	0.76
					L	100	6.15	0.61	−1.33	0.74
					J	100	6.44	0.63	−1.05	0.73
					I	100	6.23	0.63	−1.26	0.75
					CLRIS, SIRIUS					
					G	91.58	14.69	3.69	0.83	2.17
					L	74.39	16.10	4.74	1.20	4.26
					J	80.35	16.04	3.61	1.52	2.30
					I	75.79	15.85	3.52	1.27	2.51
					VDIS, SIRIUS					
					G	78.70	8.13	2.45	−0.95	1.11
					L	69.68	8.17	2.19	−1.29	1.08
					J	57.80	8.44	2.39	−0.84	1.19
					I	74.34	8.18	2.36	−1.17	1.12
					HRIS, SIRIUS					
					G	78.89	4.26	1.10	−0.13	0.85
					L	81.71	4.16	1.07	−0.22	0.88
					J	73.86	4.35	1.12	−0.08	0.80
					I	83.36	4.25	1.17	−0.14	0.96

3	[53]	An automated vessel diameter measurement technique	Retinal blood vessel width	Unsupervised Linear Discriminant Analysis Diameter Measurement	REVIEW, 5066 Profiles					
					KPIS	100	7.02	0.67	−0.50	0.60
					CLRIS	98.20	13.23	3.55	−0.55	1.79
					VDIS	96.3	8.68	2.82	−0.64	1.18
					HRIS	99.6	4.19	1.35	0.21	0.79
4	[54]	An algorithm for estimating the width of a retinal blood vessel in fundus camera images.	Retinal blood vessel width	Supervised learning is performed by bagged decision trees and an extended multiresolution Hermite model	REVIEW, 5066 Profiles					
					KPIS	100	7.54	0.24	O.015	0.318
					CLRIS	100	13.80	3.89	0.006	1.154
					VDIS	100	8.87	2.22	0.015	1.073
					HRIS	100	4.36	1.13	0.004	0.438
					Tayside data set, 38 fundus images	100	20.43	3.55	0.03	3.168

5	[24]	An algorithm to measure the width of the retinal vessels and find the vessels boundary in fundus photographs	Retinal blood vessel width	Graph-based segmentation method	REVIEW, 5066 profiles					
					KPIS	99.4	6.38	0.59	−1.14	0.67
					CLRIS	94.10	14.05	4.47	0.08	1.78
					VDIS	96.0	8.35	3.00	−0.53	1.43
					HRIS	100	4.56	1.30	0.21	0.567

6	[20]	A novel method for measuring the blood vessel diameter in the retinal image.	Retinal blood vessel width	Thresholding segmentation and thinning step, followed by Douglas-Peucker algorithm. active contours and Heron's Formula	STARE Database		7.73242	0.016	−0.01892	
							4.9278	0.1094	0.06298	
							5.39212	0.1401	0.00544	
					HRF Database		7.7316	0.0234	0.0286	
							15.17584	0.0092	0.00352	
							13.84122	0.0063	−0.00738	

7	[21]	An algorithm for the segmentation and measurement of retinal vessels width, the ESP algorithm.	Retinal blood vessel width	Active contour model	REVIEW, 5066 profiles					
					KPIS	100	6.56			0.328
					CLRIS	93.00	15.70			1.469
					VDIS	99.0	8.80			0.766
					HRIS	99.7	4.63			0.420

8	[26]	An adaptive model to measure the width of retinal vessels in fundus photographs.	Retinal blood vessel width	Adaptive Higuchi's Dimension	REVIEW, 5066 profiles		Precision		Accuracy	
					KPIS	100	0.45		0.72	
					CLRIS	98.00	1.56		0.33	
					VDIS	97.8	1.14		0.45	
					HRIS	99.4	0.65		0.24	

9	[25]	A technique of retinal vessel diameter measurement.	Retinal blood vessel width	Multi-Step Regression Method (Higher order Gaussian modeling)	REVIEW		Precision		Accuracy	
					CLRIS		1.691		−1.574	
					VDIS		1.182		−0.443	
